# Impact of the COVID-19 pandemic on training and technology use among Chilean amateur athletes

**DOI:** 10.3389/fspor.2024.1302023

**Published:** 2024-03-11

**Authors:** Natalia Chahin-Inostroza, Fanny Bracho-Milic, Edith Velasco-Bahamonde, Claudia Navarrete-Hidalgo, Pamela Serón

**Affiliations:** ^1^ Medical and Health Sciences Department, Universidad Mayor, Temuco, Chile; ^2^ Medical School, Centro de Excelencia CIGES, Universidad de La Frontera, Temuco, Chile; ^3^ Medical School, Rehabilitation Sciences Department, Universidad de La Frontera, Temuco, Chile

**Keywords:** coronavirus disease 2019, recreational athletes, physical training, running, triathletes, mobile applications, wearable technologies

## Abstract

**Introduction:**

The COVID-19 pandemic was a health problem which affected the entire world. Sports were strongly affected, especially outdoors. The purpose of this study was to evaluate the impact of COVID-19 pandemic on training and technology use among Chilean amateur athletes.

**Method:**

An observational descriptive cross-sectional study, carried out during the 2021–2. Nonprobabilistic convenience sample of people over 18 years. Data were obtained via online survey and analyzed with Stata 16.0 statistical program for runners, triathletes, cyclists.

**Results:**

The sample was 179 athletes, average age was 42.5 years ±10.2; males were 58.6%. 22.65% of the sample were triathletes, 58% runners, and 18.2% cyclists. Training habits were measured during Pre-Pandemic (PP), Pandemic With Quarantine (PWQ), and Pandemic Without Quarantine (PWOQ). In total sample, a decrease was observed in variables of average training frequency of 1.28 sessions per week (*p* = 0.001; *d* = 0.648); weekly average training time of 189.63 min (*p* = 0.005; *d* = 0.293); days per week with high and medium intensity training of 0.95 (*p* = 0.001; *d* = 0.833) and 0.37 (*p* = 0.001; *d* = 0.327) respectively; and days per week with cardio training of 1.01 (*p* = 0.001; *d* = 0.678), comparing the PP and PWQ periods. When comparing PWQ and PWOQ, an increase was observed in the same variables mentioned above of 1,57 sessions per week (*p* = 0.001; *d* = 0.513); 162.68 min per week (*p* = 0.020; *d* = −0.245); days per week with high of 0.82 (*p* = 0.001; *d* = −0.714) and medium intensity training of 0.46 (*p* = 0.001; *d* = −0.412); days per week with cardio training of 1.14 (*p* = 0.001; *d* = −0.730); and included strength training of 0.42 (*p* = 0.012; *d* = −0.312). For technology incorporation, over 78% (*p* = 0.023) claimed to used devices to measure training, with the watch being the preferred device in over 72% (*p* = 0.002) during the three timeframes. Highlighted the rise in use of training software during and after the lockdown period of more than 23% (*p* < 0.001).

**Discussion:**

All variables related with training habits decreased comparing PP and PWQ and all variables rose between PWQ and PWOQ; however, comparing PP and PWOQ, there are small differences, which do not always favor the PWOQ, reflecting how athletes have not yet been able to recover their training rhythms. Finally, we should note that the use of technology increased, in all periods.

## Introduction

In March 2020, the World Health Organization (WHO) declared a state of pandemic for COVID-19, classified as a public health emergency of international concern (1). Nations joined forces to prevent infections, resulting in industry closures and a complete lifestyle change for people due to the implementation of government rules such as circulation restrictions, distancing, and social isolation. This led to a substantial impact at all levels of daily life, including sports. The majority of historic sporting events, whether international ones such as the 2020 Tokyo Olympics and the UEFA Euro 2020 Cup, or national ones within various countries, had to be postponed or canceled (2).

In Chile, the reality was similar to the countries in the northern hemisphere, with the cancellation of massive sporting events such as the Santiago marathon, Ironman Pucón and the Temuco-Araucanía international marathon. Athletes also had to quickly modify their training habits without sufficient time to collaborate with trainers in developing structured strategies for progression (3). The suspension of competitions left athletes in a transitional phase within their training period, which could lead to a partial or total loss of adaptations arising from exercise due to insufficient stimuli, reducing maximum and sub-maximum performance in aerobic exercise within a few weeks, coinciding with deficiencies in cardiovascular function and muscle metabolism (4).

Individual amateur athletes, defined as activities pursued in leisure time, for personal satisfaction, without financial compensation (5), including triathletes, runners and cyclists were less affected during isolation phases due to the lower risk of virus spread and contagion, compared to collective or contact sports (6).

Previous studies on runners and cyclists, using an online survey methodology to collect information on their training habits (3, 7), show that they decreased their training habits at the beginning of the pandemic, but then adapted their habits to regain the previous rhythm (8).

As running consistently stands out as one of the most popular forms of exercise, due to its low cost, easy accessibility, and the social commitment arising from belonging to running clubs, with 7.9 to 13.3% of adults participating in races worldwide (9) it is interesting to study. The impact of the COVID-19 pandemic on runners training habits has been a topic of interest addressed by various research groups (3, 4, 10). Cyclists were also affected during the confinement period, mainly because they had to adapt road training to indoor settings, combined with the use of technological tools such as training planning and monitoring apps to maintain their performance (11). Given this situation, athletes must be able to incorporate diverse home training methods to maintain their physical capacity.

Additionally, in a lockdown scenario, technology becomes a fundamental tool for monitoring vital signs, socialization, follow-up, and professional advice. Athletes must restructure their planning, period division, considerations, and training load adjustments, while directives are created to return to sporting routines (9, 12). The adoption of advanced technologies entails a considerable monetary cost, including investment in specialized equipment, customized software, and high-speed connectivity.

Most of these reports are from Europe and North America (3, 8, 10, 11, 13–15), therefore, it becomes interesting to explore how athletes modified their training habits during different pandemic timeframes in countries in the southern hemisphere such as Chile, mainly because the pandemic approach and restrictions varied from one country to another. In Chile, the government established a “step-by-step” plan, in which, according to different indicators of epidemiological and health variables as well as the capacity of the healthcare network, each region and municipality transitioned through phases. These phases ranged from total quarantine, which consisted of strict confinement, to “transition”, “preparation”, and the “initial opening” phase, allowing activities to be carried out outside the home with established limits (16).

In this context, the present study aims to evaluate the impact of the COVID-19 pandemic on training and technology use among Chilean amateur athletes, particularly among triathletes, runners, and cyclists, across three different timeframes: pre-pandemic, pandemic with quarantine, and pandemic without quarantine.

## Materials and methods

### Design

A descriptive, observational, cross-sectional study was done during the years 2021–2022.

### Participants

The participants were adult Chilean amateur runners, triathletes, and cyclists from around the country, who usually participated in local, national, or international competitions in their respective disciplines.

The selection criteria for the sample were people over age 18, who did individual sports such as running, cycling, or triathlon; with over 1 year of experience or regular training; minimum training of 3 days/week or 5 h/week; intellectually capable of answering the survey and accepting participation in the study by signing informed consent.

In the invitation to participate, the selection criteria for the study were clearly detailed, so that only those who met them were eligible to participate in the survey.

The sample included all the subjects who answered the self-reported online survey during the months of April and May 2022.

The study was approved by the Scientific Ethics Committee of the *Servicio de Salud Araucanía Sur* in the city of Temuco, Chile.

A non-probabilistic convenience sample of all persons interested in answering the online survey was used. The survey was disseminated and published via a link on the official page of the Communications Platform for RUN Chile, which includes runners, and TRI Chile which includes triathletes, and cyclists. The study was also published with its respective survey link on Instagram, and the athletes themselves were asked to spread the link among people who competitively practiced any of the sports mentioned. This was available for response during 2 months.

### Variables and measurements

Data were obtained via a self-applied online survey which took around 15 min to complete, created by the researchers, and which underwent face and content validation by external expert judges (Appendix 1).

The survey included a total of 29 short-answer questions, with both quantitative and qualitative indicators, which feed into a 4-dimensional comprehension: sociodemographic aspects, training habits, training budget, and technology use.

The dimensions of the survey corresponding to training habits, training budget, and technology use were evaluated by external judges. The validity of each item or question was appraised via three categories: “essential for dimension evaluation”, “useful but disposable”, and “unnecessary”. Global evaluation of each dimension was also evaluated via its “sufficiency”, considering whether the list of questions comprising the dimension were enough to obtain its value (17) (Appendix 2).

The profile defined for selecting expert external judges considered the inclusion of professionals in sports and training; along with competitive athletes participating in individual disciplines such as running, cycling, and triathlon. A methodologist was also included to evaluate the survey structure.

The study variables for each survey dimension were as follows:

In sociodemographic aspects, data was gathered about birthdate (in years), biological sex (with the response options of male, female, or other), highest finished educational level (from No Formal Studies to postgraduate level), socioeconomic level by total monthly household income (which was later used to classify the socioeconomic level at Upper class: ABC1 level; middle class: C1b, 2, and 3 levels; lower class: D and E levels), origin (urban, rural), occupation (employer, independent worker, public sector employee, private sector employee, domestic service, uncompensated family work, military or police, housewife, not employed) and type of sport presented as the response options of running, cycling, swimming, and triathlon. For this variable, the instructions specified that triathletes should select only this option, and not each of the previous separately. Finally, one last variable was added corresponding to the time spent systematically carrying out their training routine (in years and months), before pandemic.

For the dimensions of training habits, training budget, and technology use, questions were asked based on 3 different timeframes considering stages Pre-Pandemic (PP), Pandemic With Quarantine (PWQ), and Pandemic Without Quarantine (PWOQ).

For training habits, questions focused on average training frequency (reported as training sessions per week); average duration of weekly training (in min/week); number of days per week with high-intensity training (80%–90% Maximum Heart Rate (MHR) or >85% 1 Maximum Repetition (MR); number of days per week with moderate-intensity training (70%–80% MHR or 60%–85% 1MR); and number of days per week with low-intensity training (60%–70% MHR or 30%–60% 1MR) (18); primary training location described as home, gym, urban outdoors (bike paths, plazas, parks) and nature (hills, national parks), with more than one response option available; number of days per week with cardio and strength training, to be answered in a discreet quantitative form.

For training budgets, the questions focused on roughly how much money was spent on average each month for training, considering the items of gym fees, trainers, and health professionals. We also asked about the amount spent on equipment for training during the period 2019–2021, considering monitoring devices, machine equipment, training software, sportswear, and nutritional supplements.

For technology use, dichotomous questions were presented about the use of specialized software for training; the use of devices to measure training parameters, and the use of training practice equipment. For affirmative answers, respondents were asked which things they used.

Data gathering was done via an online platform and dumped into an anonymized database for management and analysis.

### Statistical analysis

The Stata v.16.0 statistical program was used for the data analysis process. The group description was done as a function of the athletic disciplines: runners, triathletes and cyclists, considering the 4 domains comprising the survey. Adequate Descriptive Statistics tools were used for each type of variable. To describe the sociodemographic characteristics of the sample, relative and absolute frequency measurements were used for the categorical variables [sex, Educational Level (EDL), socioeconomic level, region, origin, occupation], while for the numerical variables (age and time spent doing the sport/discipline), central trend measurements such as the median were used, while the standard deviation was used for dispersion. The Chi-squared statistical test was applied for categorical variables to evaluate that differences between groups were statistically significant, considering a value of *p* < 0.05.

The homogeneity test was performed using the Shapiro-wilk test. Parametric variables were evaluated by repeated measures ANOVA for quantitative variables by discipline (runners, triathletes, cyclists), with Sidak *post hoc* analysis, as appropriate. The variables for which the parametric test was applied were age, average training frequency in PP, days/week Cardio training in PP and PWOQ. The Kruskall–Wallis test was used for the nonparametric variables, which corresponded to all the others.

An intra-group analysis was done comparing 3 timeframes: pre-pandemic, pandemic with quarantine, and pandemic without quarantine. *T*-tests were done in each of the groups (disciplines).

Moreover, the effect size, Cohen's was calculated for all variables with the thresholds for small, moderate, and large effects set to 0.20, 0.50, and 0.80, respectively (19).

## Results

One hundred and seventy-nine athletes were included, of which 58,6% were runners, 22.9% were triathletes, and 18.4% were cyclists. The average age was 42.5 years ±10.2, with 58.6% men; high EDL (77.4%), of which 37.6% declared they had graduated from university and 51.4% reported postgraduate studies. Socioeconomic Level presented a predominantly middle (C1b, C2, C3) and upper-class distribution (AB, C1a), at 52.1% and 36.3% respectively. For the geographical distribution, 94.5% lived in urban areas, with a majority residing in the Metropolitan Region (50.8%) over the northern, central, and southern zones. Private sector workers and employees were the primary occupation group, at 42.5%. Finally, the average time since respondents began to do their sport was 7 years and 11 months. The details by discipline appear in Table 1.

**Table 1:** Sociodemographic characteristics by discipline.

Sociodemographic characteristics	Total (*n* = 179)	Triathletes (*n* = 41)	Runners (*n* = 105)	Cyclists (*n* = 33)	*p*-Value
Age (avg ± SD)	42.5 ± 10.2	42.4 ± 10.1	42.1 ± 10.3	42.7 ± 10.2	0.129
Sex (%, *n*)					
Female	41.4% (75)	36.6% (15)	52.4% (55)	15.2% (5)	0.001
Male	58.6% (106)	63.4% (26)	47.6% (50)	84.8% (28)	
Ed. level (%, *n*)					
Secondary school graduate	3.3% (6)	2.4% (1)	4.8% (5)	0.0% (0)	0.623
Secondary school, incomplete	0.6% (1)	0.0% (0)	1.0% (1)	0.0% (0)	
Primary school graduate	0.6% (1)	0.0% (0)	1.0% (1)	0.0% (0)	
Technical/profesional school graduate	3.9% (7)	0.0% (0)	5.7% (6)	3.0% (1)	
Technical/profesional school, incomplete	2.2% (4)	0.0% (0)	1.9% (2)	6.1% (2)	
Postgraduate	39.8% (72)	36.6% (15)	35.2% (37)	54.6% (18)	
University graduate	37.6% 68)	46.3% (19)	36.2% (38)	33.3% (11)	
University dropout	12.2% (22)	14.6% (6)	14.3% (15)	3.0% (1)	
Socioeconomic level (%, *n*)					
AB	11.5% (19)	9.7% (3)	9.8% (10)	16.1% (5)	0.110
C1a	24.8% (41)	38.7% (12)	16.7% (17)	38.7% (12)	
C1b	18.2% (30)	6.5% (2)	21.6% (22)	19.4% 6)	
C2	21.8% (36)	19.4% (6)	25.5% (26)	12.9% (4)	
C3	12.1% (20)	19.4% (6)	11.8% (12)	6.5% (2)	
D	4.9% (8)	3.2% (1)	5.9% (6)	3.2% (1)	
E	6.7% (11)	3.2% (1)	8.8% (2)	3.2% (1)	
Zone					
North	6.1% (11)	9.8% (4)	3.8% (4)	9.1% (3)	0.534
Center	19.9% (36)	21.9% (9)	20.9% (22)	15.2% (5)	
Metropolitan	50.8% (92)	36.6% (15)	54.3% (57)	57.6% (19)	
South	23.2% (42)	31.7% (13)	20.9% (22)	18.2% (6)	
Origin (%, *n*)					
Rural	5.5% (10)	7.3% (3)	4.8% (5)	6.1% (2)	0.918
Urban	94.5% (171)	92.7% (38)	95.2% (100)	93.9% (31)	
Occupation (%, *n*)					
Housewife	3.3% (6)	2.4% (1)	3.8% (4)	3.0% (1)	0.934
Public enterprise worker or employee	10.5% (19)	12.2% (5)	9.5% (10)	12.1% (4)	
Private sector worker or employee	42.5% (77)	31.7% (13)	45.7% (48)	45.5% (15)	
Public sector employee and worker	8.8% (16)	12.2% (5)	8.6% (9)	6.1% (2)	
Military or police	3.3% (6)	4.9% (2)	3.8% (4)	0.0% (0)	
Unemployed	4.4% (8)	2.4% (1)	5.7% (6)	3.0% (1)	
Employer or business owner	3.9% (7)	7.3% (3)	1.0% (1)	9.1% (3)	
Domestic service	1.1% (2)	0.0% (0)	1.9% (2)	0.0% (0)	
Self-employed	22.1% (40)	26.8% (11)	20.0% (21)	21.2% (7)	
Private sector employee or worker	42.5% (77)	31.7% (13)	45.7% (48)	45.5% (15)	
Public sector worker and employee	8.8% (16)	12.2% (5)	8.6% (9)	6.1% (2)	
Military and police	3.3% (6)	4.9% (2)	3.8% (4)	0.0% (0)	
Unemployed	4.4% (8)	2.4% (1)	5.7% (6)	3.0% (1)	
Employer or business owner	3.9% (7)	7.3% (3)	1.0% (1)	9.1% (3)	
Domestic service	1.1% (2)	0.0% (0)	1.9% (2)	0.0% (0)	
Self-employed	22.1% (40)	26.8% (11)	20.0% (21)	21.2% (7)	
Time practicing sport	7.9 ± 6.60 (7 years and 11 months)	6.5 ± 5.57 (6 years, 6 months)	7.8 ± 6.15 (7 years, 10 months)	9.4 ± 7.85 (9 years, 5 months)	0.084

About training habits, when comparing PP and PWQ periods, a significant (*p* < 0.001) decrease was observed in the average weekly training frequency, average weekly training time, days per week with high and medium intensity training, days per week with cardiovascular and strength training (*p* = 0.012). The same variables showed a significant (*p* < 0.001) rise when comparing the PWQ and PWOQ periods. Highlights the effect size moderate for average training frequency (*d* = 0.648; *p* < 0.001 and *d* = 0.513; *p* < 0.001) and days per week cardio training (*d* = 0.678; *p* < 0.001 and *d* = −0.730, *p* < 0.001), and large/moderate for days per week with high intensity training (*d* = 0.833; *p* < 0.001 and *d* = 0.714; *p* < 0.001) between PP with PWQ, and between PWQ with PWOQ respectively. Finally, the predominant training site showed that 40.24% preferred training in urban outdoor spaces PP, similar to the PWOQ period at 39.1%. During PWQ 70.06% declared their home was the main training site (*p* < 0.001) (Table 2). A similar trend appeared when analyzing samples by sport, as shown in Table 3. Table 4 shows the changes in training habits by discipline, according to timeframe. For the rest of the comparisons, there were no significant differences.

**Table 2:** Total training habits, by timeframe.

	Pre pandemic	Total		*p*-Value	Delta 1 MD (CI)	*p*-Value	*d*	Delta 2 MD (CI)	*p*-Value	*d*
		With quarantine	Without quarantine							
Average training frequency	4.74 ± 1.73 (4.47–5.00)	3.46 ± 2.19 (3.13–3.79)	5.03 ± 3.73 (4.47–5.64)	<0.001	1.28 (0.86–1.69)	0.001	0.648	−1.57 (−2.20 to 0.93)	0.001	0.513
Average weekly training time	443.54 ± 783.28 (326.67–560.407)	253.91 ± 473.06 (183.33–324.49)	416.59 ± 802.54 (295.80–537.38)	<0.001	189.63 (55.12–324.12)	0.005	0.293	−162.68 (−299.61 to −25.74)	0.020	−0.245
Days/week with HIGH intensity training	2.21 ± 1.21 (2.03–2.39)	1.26 ± 1.37 (1.05–1.49)	2.09 ± 1.23 (1.89–2.27)	<0.001	0.95 (0.68–1.21)	0.001	0.833	−0.82 (−1.00 to −0.63)	0.001	−0.714
Days/week with MEDIUM intensity training	2.04 ± 1.21 (1.86–2.22)	1.67 ± 1.40 (1.46–1.88)	2.14 ± 1.26 (1.04–2.34)	<0.001	0.37 (0.17–0.57)	0.001	0.327	−0.46 (−0.64 to 0.28)	0.001	−0.412
Days/week with LOW intensity training	1.04 ± 1.08 (0.88–1.21)	1.01 ± 1.25 (0.81–1.19)	1.15 ± 1.23 (0.96–1.34)	0.604	0.04 (−0.11 to 0.19)	0.604	0.039	−0.13 (−0.27 to 0.01)	0.064	−0.120
Main training location										
Undeclared	0.61% (1)	7.19% (12)	1.92% (3)	<0.001						
Home	19.51% (32)	70.06% (117)	31.41% (49)							
Gym	23.78% (66)	2.40% (4)	8.97% (14)							
Urban open air	40.24% (66)	13.17% (22)	39.10% (61)							
Nature	15.85% (26)	7.319% (12)	18.59% (29)							
Days/week Cardio training	3.50 ± 1.79 (3.19–3.77)	2.49 ± 1.95 (2.18–2.79)	3.61 ± 1.87 (3.29–3.89)	<0.001	1.01 (0.69–1.34)	0.001	0.678	−1.14 (−1.40 to 0.89)	0.001	−0.730
Days/week Strength training	1.66 ± 1.49 (1.42–1.89)	1.41 ± 1.41 (1.41–1.63)	1.85 ± 2.05 (1.51–2.15)	0.012	0.24 (−0.03 to 0.51)	0.081	0.230	−0.42 (−0.75 to 0.09)	0.012	−0.312

Delta 1: Compared Pre-Pandemic (PP) vs. Pandemic With Quarantine (PWQ).

Delta 2: Compared Pandemic With Quarantine (PWQ) vs. Pandemic Without Quarantine (PWOQ).

MD, mean difference; CI, confidence interval; *d*, Cohen's *d*, Effect size.

**Table 3:** Training habits by discipline, according to timeframe.

	Pre pandemic	Triathletes		*P*-value	Pre-pandemic	Runners		*p*-Value	Pre-pandemic	Cyclists		*p*-Value
		With quarantine	Without quarantine			With quarantine	Without quarantine			With quarantine	Without quarantine	
Avg training frequency	5.55 ± 1.76 (4.97–6.13)	4.26 ± 2.15 (3.55–4.97)	6.86 ± 6.80 (4.67–9.37)	0.030	4.54 ± 1.73 (4.25–4.94)	3.24 ± 2.16 (2.81–3.67)	4.65 ± 1.83 (4.27 −5.02)	<0.001	4.26 ± 1.43 (3.82–4.85)	3.36 ± 2.07 (2.45–3.97)	4.08 ± 1.46 (3.53–4.62)	0.027
Avg weekly training time	240.59 ± 1,521.64 (296.35–1,269.00)	142.73 ± 902.70 (123.05–700.44)	775.72 ± 1,559.16 (277.08–1,274.00)	0.050	288.51 ± 253.08 (238.55–338.47)	179.65 ± 188.61 (142.41–216.88)	277.82 ± 250.77 (227.81–327.84)	<0.001	508.12 ± 302.38 (399.10–617.14)	288.12 ± 242.70 (200.62–375.62)	403.87 ± 250.88 (311.84–495.89)	0.005
Days/wk with HIGH intensity training	2.39 ± 1.53 (1.89–2.89)	1.42 ± 1.36 (0.97–1.87)	2.29 ± 1.39 (1.88–2.92)	0.001	2.22 ± 1.14 (1.99–2.45)	1.22 ± 1.41 (0.94–1.50)	2.12 ± 1.19 (1.88–2.36)	<0.001	2.06 ± 0.91 (1.73–2.39)	1.25 ± 1.29 (0.78–1.71)	1.80 ± 1.09 (1.39–2.20)	0.015
Days/wk with MEDIUM intensity training	2.34 ± 1.36 (1.89–2.78)	2.00 ± 1.59 (1.47–2.52)	2.35 ± 1.47 (1.86–2.78)	0.011*	2.01 ± 1.13 (1.78–2.23)	1.61 ± 1.33 (1.34–1.88)	2.22 ± 1.13 (1.99–2.46)	0.044	1.87 ± 1.21 (1.43–2.31)	1.50 ± 1.34 (1.01–1.98)	1.70 ± 1.31 (1.20–2.19)	0.432
Days/wk with LOW intensity training	1.28 ± 133 (0.85–1.72)	1.21 ± 1.35 (1.35–1.65)	1.27 ± 1.32 (0.84–1.74)	0.623	0.92 ± 0.83 (0.76–1.09)	0.92 ± 1.17 (0.67–1.15)	1.13 ± 1.17 (0.88–1.36)	0.04**	0.93 ± 1.16 (0.52–1.35)	1.00 ± 1.34 (0.51–1.48)	1.06 ± 1.31 (0.57–1.55)	0.677
Main training site												
Undeclared	0.00% (0)	5.26% (2)	2.70% (1)	0.04***	1.09% (1)	6.19% (6)	1.12 (1)	<0.001	0.00% (0)	10.00% (3)	0.00% (0)	<0.001**
Home	28.21% (11)	81.58% (31)	45.95% (17)		14.13% (13)	68.04% (66)	24.72% (22)		22.58% (7)	63.33% (19)	32.14% (9)	
Gym	17.95% (7)	5.26% (2)	10.81% (4)		28.26% (26)	1.03% (1)	10.11% (9)		16.13% (5)	3.33% (1)	3.57% (1)	
Urban outdoors	48.72% (19)	7.89% (3)	29.73% (11)		43.48% (40)	17.53% (17)	48.31% (42)		22.58% (7)	6.67% (2)	25.00% (7)	
Nature	5.13% (2)	0.00% (0)	10.81% (4)		13.04% (12)	7.22% (7)	15.73% (14)		38.71 (12)	16.67% (5)	39.29% (11)	
Days/wk Cardio training	4.08 ± 2.26 (3.32–4.83)	3.08 ± 2.28 (2.31–3.844)	4.58 ± 2.33 (3.28–4.83)	0.014	3.53 ± 1.58 (3.21–3.86)	2.35 ± 1.85 (1.97–2.73)	3.58 ± 1.57 (3.25–3.91)	<0.001	2.74 ± 1.56 (2.16–3.31)	2.29 ± 1.73 (1.65–2.92)	2.65 ± 1.54 (2.02–3.17)	0.630
Days/wk Strength training	1.64 ± 2.00 (0.98–2.31)	1.56 ± 1.14 (1.18–1.94)	1.44 ± 0.93 (1.12–1.76)	0.827	1.74 ± 1.41 (1.45–2.04)	1.48 ± 1.58 (1.15–1.81)	2.16 ± 2.52 (1.61–2.67)	0.018*	1.53 ± 0.97 (1.14–1.85)	1.46 ± 1.10 (0.66–1.53)	1.50 ± 1.13 (1.05–1.87)	0.782

* Difference only between Pandemic With and Without Quarantine (no difference in the others).

** Difference only between Pre-Pandemic and Pandemic Without Quarantine (no difference in the others).

*** Difference between Pre-Pandemic with Pandemic without quarantine and Pandemic With and Without Quarantine.

**Table 4:** Changes in training habits by discipline, according to timeframe.

	Delta 1 MD (CI)	Triathletes	*d*	Delta 2 MD (CI)	*p*-Value	*d*	Delta 1 MD (CI)	Runners	*d*	Delta 2 MD (CI)	*d*	*p*-Value	Delta 1 MD (CI)	Cyclists	*d*	Delta 2 MD (CI)	*p*-Value	*d*
		*p*-Value						*p*-Value						*p*-Value				
Avg training time	1.28 (0.38–2.19)	0.006	0.826	−2.68 (−5.09 to −0.27)	0.030	−0.558	1.29 (0.80–1.78)	<0.001	0.847	−1.43 (−1.73 to −1.12)	0.452	<0.001	1.12 (0.13–2.11)	0.027	0.845	−0.71 (−1.27 to −0.15)	0.013	−0.474
Avg weekly training time	371.25 (−0.15 to 742.65)	0.050	0.343	−363.97 (−731.91 to 3.96)	0.052	−0.346	108.86 (57.01–160.71)	<0.001	0.500	−100.90 (−135.71 to −66.10)	−0.582	<0.001	220.00 (70.31–369.68)	0.005	0.718	−106.45 (−180.66 to −32.23)	0.006	−0.309
Days/wk with HIGH intensity training	0.97 (0.42–1.52)	0.001	0.808	−0.89 (−1.21 to 0.56)	0.001	−0.757	1.00 (0.65 to 1.34)	<0.001	0.779	−0.92 (−1.19 to −0.66)	−0.851	<0.001	0.81 (0.016–1.45)	0.015	0.808	−0.46 (−0.86 to −0.06)	0.024	−0.428
Days/wk with MEDIUM intensity training	0.34 (−0.04 to 0.73)	0.085	0.282	−0.37 (−0.66 to −0.09)	0.011	−0.305	0.39 (0.11–0.67)	0.006	0.361	−0.60 (−0.87 to −0.34)	−0.518	<0.001	0.375 (−0.13 to 0.88)	0.142	0.332	−0.16 (−0.47 to 0.14)	0.432	−0.144
Days/wk with LOW intensity training	0.07 (−0.17 to 0.33)	0.538	0.068	−0.05 (−0.27 to 0.16)	0.623	−0.046	0.01 (−0.21 to 0.23)	0.926	0.010	−0.18 (−0.40 to 0.02)	−0.175	0.077	−0.06 (−0.32 to 0.19)	0.624	−0.056	−0.06 (−0.39 to 0.25)	0.677	−0.057
Days/wk Cardio training	1.00 (0.21–1.78)	0.014	0.441	−1.55 (−2.21 to −0.89)	0.001	−0.684	1.18 (0.75–1.61)	<0.001	0.798	−1.25 (−1.58 to −0.93)	−0.863	<0.001	0.45 (−0.17 to 1.07)	0.151	0.304	−0.37 (−0.87 to 0.11)	0.125	−0.268
Days/wk Strength training	0.08 (−0.66 to −0.82)	0.827	0.054	0.08 (−0.29 to 0.45)	0.653	0.091	0.26 (−0.08 to 0.61)	0.139	0.215	−0.66 (−1.21 to −1.14)	−0.436	0.018	0.40 (−0.10 to 0.90)	0.116	0.391	−0.35 (−0.72 to 0.01)	0.057	−0.332

Delta 1: Compared Pre-Pandemic (PP) vs. Pandemic With Quarantine (PWQ).

Delta 2: Compared Pandemic With Quarantine (PWQ) vs. Pandemic Without Quarantine (PWOQ).

MD, mean difference; CI, confidence interval; *d*, Cohen's *d*, Effect size.

After considering the monthly training budget measured in 3 different timeframes (PP, PWQ, and PWOQ) among the total sample of athletes, statistically significant differences only appeared in the budget for trainers, both among triathletes (*p* = 0.011) and runners (*p* = 0.011), showing a slight rise during the PWQ and PWOQ periods compared to PP; budget for health professionals for runners (0.001), showing a rise during the PWOQ compared to PWQ; and budget for gym in cyclist (*p* < 0.001) with a decrease between PP and PWOQ (Table 5). Changes in the monthly training budget by discipline are shown in Table 6, which highlights an increase in the budget for health professional in triathletes (*d* = −1.556; *p* = 0.043) between PWQ and PWOQ.

**Table 5:** Monthly training budget, by discipline.

Training budget/Monthly	Triathletes				Runners				Cyclists			
	Pre-pandemic	With quarantine	Without quarantine	*p*-Value	Pre-pandemic	With quarantine	Without quarantine	*p*-Value	Pre-pandemic	With quarantine	Without quarantine	*p*-Value
Gym	59.69 ± 49.02 (1.18–120.56)	57.43 ± 44.42 (2.92–112.58)	54.99 ± 35.98 (32.13–77.86)	0.485	59.69 ± 49.02 (1.18–120.56)	57,43 ± 44,41 (2,92–112,58)	54,99 ± 35,98 (32,13–77,86)	0.485	40.09 ± 19.53 (15.84–64.34)	2.25 (2.25–2.25)	30.40 ± 9.42 (18.70–42.10)	0.001
Trainer	57.38 ± 26.26 (45.42–69.37)	62.90 ± 31.77 (48.45–77.35)	71.03 ± 36.09 (55.02–87.03)	0.011	57.38 ± 26.26 (45.42–69.37)	62,90 ± 31,77 (48,45–77,35)	71,03 ± 36,09 (55,02–87,03)	0.011	69.68 ± 45.64 (31.52–107.84)	68.27 ± 47.05 (28.93–10.62)	64.44 ± 45.49 (29.47–99.41)	0.346
Health Professionals	31.91 ± 7.69 (23.83–39.98)	35.19 ± 18.99 (15.26–55.12)	49.03 ± 38.71 (26.67–71.38)	0.286	65.69 ± 7.69 (23.83–55.12)	35.19 ± 18.99 (15.26–55.12)	49.03 ± 38.71 (26.67–71.38)	0.001	48.26 ± 32.97 (11.26–11.26)	50.68 ± 39.81 (22.52–78.83)	101.36 ± 92.18 (22.52–202.72)	0.644

Value in USD, converted from Chilean peso (20/09/2023) according to Banco Central Chile.

**Table 6:** Changes in monthly training budget, by discipline.

Training budget/Monthly	Triathletes						Runners						Cyclists					
	Delta 1 MD (CI)	*p*-Value	*d*	Delta 2 MD (CI)	*p*-Value	*d*	Delta 1 MD (CI)	*p*-Value	*d*	Delta 2 MD (CI)	*p*-Value	*d*	Delta 1 MD (CI)	*p*-Value	*d*	Delta 2 MD (CI)	*d*	*p*-Value
Gym	2.26 (−18.29 to 22.81)	0.827	0.039	2.44 (−15.32 to 20.20)	0.785	0.093	2.26 (−10.46 to 14.98)	0.726	−0.067	2.44 (−8.55 to 13.43)	0.662	0.002	37.84 (34.37–41.30)	0.001	-	−28.15 (−31.57 to 24.72)	-	0.001
Trainer	−5.52 (−18.33 to 7.29)	0.393	−0.191	−8.13 (−23.07 to 6.81)	0.282	−0.267	−5.52 (−13.45 to 2.41)	0.171	0.154	−8.13 (−17.38 to 1.12)	0.084	0.217	1.41 (−21.75 to 24.57)	0.903	0.022	3.83 (−19.29 to 26.95)	0.082	0.741
Health Professionals	−3.28 (−9.64 to 3.08)	0.308	−0.210	−13.84 (−27.24 to −0.43)	0.043	−1.556	30.50 (26.57–34.44)	<0.001	0.296	−13.84 (−22.13 to −5.54)	0.001	0.221	−2.42 (−20.68 to 15.84)	0.792	−0.080	−50.68 (−86.16 to −15.19)	−0.606	0.058

Delta 1: Compared Pre-Pandemic (PP) vs. Pandemic With Quarantine (PWQ).

Delta 2: Compared Pandemic With Quarantine (PWQ) vs. Pandemic Without Quarantine (PWOQ).

MD, mean difference; CI, confidence interval; *d*, Cohen's *d*, Effect size.

The amount spent on training equipment during the 2019–2021 period showed statistically significant differences in the total sample for monitoring devices, it increased considerably in PWQ (*d* = −10.76; *p* < 0.001) and then decreased in PWOQ (*d* = 2.023; *p* < 0.001) reaching levels similar to PP; equipment-machinery presenting a decrease during PWQ (*d* = 0.572; *p* < 0.001), followed by a final rise during PWOQ (*d* = 0.757; *p* < 0.001), compared to the previous; for sportswear, presenting a slight rise during the PWQ and PWOQ periods (*d* = 0.191; *p* < 0.001), without achieving the PP (Table 7).

**Table 7:** Budget for training equipment during period from 2019 to 2021, total sample.

Training budget/period	Pre-pandemic	With quarantine	Without quarantine	*p*-Value	Delta 1 MD (CI)	*p*-Value	*d*	Delta 2 MD (CI)	*p*-Value	*d*
Monitoring devices	231.89 ± 216.42 (160.75–303.02)	1,977.26 ± 151.24 (130.70–264.81)	201.24 ± 168.53 (1,458.50–256.64)	0,001	−1,745.37 (−1,484 to −1,706.55)	<0.001	−10.76	1,776.02 (1,742.73–1,809.30)	0.001	2.023
Equipment—machinery	669.10 ± 200.56 (66.56–1,404.79)	391.64 ± 652.21 (152.41–630.88)	753.65 ± 157.37 (89.11–1,418.20)	0,001	277.46 (177.15–377.76)	<0.001	0.572	−362.01 (−460.63 to −263.38)	0.001	−0.757
Training software	73.20 ± 84.89 (19.26–127.14)	81.18 ± 93.25 (21.92–14.04)	117.39 ± 243.57 (6.52–228.27)	0.296	−7.98 (−26.51 to 10.55)	0.397	−0.091	−36.21 (−74.54 to 2.12)	0.064	−0.188
Sportswear	207.63 ± 455.23 (119.53–295.74)	142.89 ± 146.91 (108.61–177.17)	177.131 ± 180.750 (151.65–247.33)	0.018	64.74 (−5.57 to 135.05)	0.071	0.195	118.90 (81.99–155.80)	<0.001	0.191
Nutritional Supplements	119.76 ± 190.79 (72.48–167.04)	98.76 ± 167.78 (57.18–140.33)	129.74 ± 214.14 (78.30–181.19)	0.128	21.00 (−16.34 to 58.34)	0.296	0.124	−30.98 (−70.96 to 9.00)	0.128	−0.163

Value in USD, converted from Chilean peso (20/09/2023) according to banco central Chile.

Delta 1: Compared Pre-Pandemic (PP) vs. Pandemic With Quarantine (PWQ).

Delta 2: Compared Pandemic With Quarantine (PWQ) vs. Pandemic Without Quarantine (PWOQ).

MD, mean difference; CI, confidence interval; d, Cohen's *d*, effect size.

The use of training measurement devices is widely practiced among amateur athletes, since when considering the 3 timeframes in the total sample, over 78% of respondents said they used them, with watches predominating over other devices. With using equipment for training practice there was a notable rise during the PWQ and PWOQ periods in the general sample (*p* < 0.001) With training software there is a growth trend between PP and the PWQ and PWOQ periods; however, when comparing PWQ and PWOQ, there is a slight decrease in the general sample (*p* < 0.001) (Table 8). Details by discipline appear in Table 9.

**Table 8:** Use of technological training tools, total sample.

Technological tool use	Total			*p*-Value
	Pre-pandemic	With quarantine	Without quarantine	
Training measurement devices				
No	14.54% (24)	21.82% (31)	5.80% (9)	0.023
Yes	85.45% (141)	78.18% (129)	94.19% (146)	
Device type used				
Watch	88.65% (125)	72.09% (93)	76.02% (111)	0.002
Band	8.51% (12)	17.05% (22)	18.49% (27)	
Cell phones and other devices	2.83% (4)	10.85% (14)	5.47% (8)	
Training practice equipment				
No	48.10% (76)	32.48% (51)	32.00% (48)	<0.001
Yes	51.89% (82)	67.51% (106)	68.00% (102)	
Equipment used for training practice				
Treadmill	28.04% (23)	21.49% (23)	18.62% (19)	<0.001
Bicycle	15.85% (13)	31.77% (34)	14.70% (15)	
Knee pads	42.68% (35)	29.90% (32)	47.05% (48)	
Weights and other equipment	13.41% (11)	15.88% (17)	19.60% (20)	
Training software				
No	68.15% (107)	41.13% (65)	44.73% (68)	<0.001
Yes	31.84% (50)	58.86% (93)	55.26% (84)	
Types of software used				
Applications	26.11% (41)	32.48% (51)	44.07% (67)	<0.001
Videoconferences (Zoom, Meet)	4.45% (7)	24.20% (38)	9.86% (15)	
Videos (YouTube or others)	1.27% (2)	2.54% (4)	1.31% (2)	

For this test *χ*^2^ was used to determine association.

**Table 9:** Technological tool use for training, by discipline.

Technological tool use	Triathletes				Runners				Cyclists			
	Pre-pandemic	With quarantine	Without quarantine	*p*-Value	Pre-pandemic	With quarantine	Without quarantine	*p*-Value	Pre-pandemic	With quarantine	Without quarantine	*p*-Value
Training measurement devices												
No	5.26% (2)	5.41% (2)	2.63% (1)	0.255	18.94% (18)	25.27% (23)	6.98% (6)	0.02	12.50% (4)	18.75% (6)	6.45% (2)	0.355
Yes	94.70% (36)	94.59% (35)	97.37% (37)		81.05% (77)	74.73% (68)	93.02% (80)		87.50% (28)	81.25% (26)	93.55% (29)	
Device type used												
Watch	86.11% (31)	54.28% (19)	59.45% (22)	0.632	93.50% (72)	85.29% (58)	91.25% (73)	0.032	78.57% (22)	61.53% (16)	55.17% (16)	0.009
Band	13.88% (5)	37.14% (13)	40.54% (15)		2.59% (2)	7.35% (5)	5.00% (4)		17.85% (5)	15.38% (4)	27.58% (8)	
Cell phones and other devices	0.00% (0)	8.57% (3)	0.00% (0)		3.89% (3)	7.35% (5)	3.75% (3)		3.57% (1)	23.07% (6)	17.24% (5)	
Training practice equipment												
No	21.05% (8)	5.71% (2)	8.33% (3)	0.047	61.80% (55)	45.55% (41)	45.24% (38)	0.011	41.94% (13)	25.00% (8)	26.67% (8)	0.016
Yes	78.95% (30)	94.29% (33)	91.67% (33)		38.20% (34)	54.44% (49)	54.76% (46)		58.06% (18)	75.00% (24)	73.33% (22)	
Equipment used for training practice												
Treadmill	16.66% (5)	15.15% (5)	18.18% (6)	<0.001	50.00% (17)	30.00% (15)	20.08% (12)	0.003	5.55% (1)	12.50% (3)	4.54% (1)	0.037
Bicycle	10.00% (3)	12.12% (4)	6.06% (2)		26.47% (9)	20.00% (10)	21.73% (10)		5.55% (1)	83.33% (20)	13.63% (3)	
Kneepads	63.33% (19)	60.60% (20)	60.60% (20)		5.88% (2)	22.00% (11)	19.56% (9)		77.77% (14)	4.16% (1)	81.81% (18)	
Weights and other equipment	10.00% (3)	12.12% (4)	15.15% (5)		17.64% (6)	26.00% (13)	32.60% (15)		11.11% (2)	0.00% (0)	0.00% (0)	
Training software												
No	52.63% (20)	21.62% (8)	26.31% (10)	<0.001	78.41% (69)	48.88% (44)	58.33% (49)	0.05	58.06% (18)	41.93% (13)	30.00% (9)	0.024
Yes	47.36% (18)	78.37% (29)	73.68% (28)		21.59% (19)	51.11% (46)	41.66% (35)		41.93% (13)	58.06% (18)	70.00% (21)	
Software type used												
Applications	77.77% (14)	44.82% (13)	71.42% (20)	0.004	78.94% (15)	56.52% (26)	88.57% (31)	<0.001	92.30% (12)	66.66% (12)	76.19% (16)	0.034
Videoconference (Zoom, Meet)	16.66% (3)	55.17% (16)	28.57% (8)		15.78% (3)	34.78% (16)	5.71% (2)		7.69% (1)	33.33% (6)	23.80% (5)	
Videos (YouTube or others)	5.55% (1)	0.00% (0)	0.00% (0)		5.26% (1)	8.69% (4)	5.71% (2)		0.00% (0)	0.00% (0)	0.00% (0)	

*χ*^2^ was used to determine the association.

## Discussion

The health crisis and lockdowns affected regular training practices. To our knowledge, this is the first report on the impact of the pandemic on training habits in amateur athletes in Chile.

As in previous similar studies, the sociodemographic characteristics of the sample coincide in the average age, medium to high educational level and mainly male participants (8, 11, 15). There could be a correlation between this profile and higher-level sports activities.

Most significant differences in training habits among all the athletes involved, as well as when broken down by discipline, were found by comparing variables in the pre-pandemic periods and the periods during the pandemic with quarantine. It should be noted that within the state of the art, no studies were found which compared three timeframes. The discussion is thus based on analyzing pre-pandemic data and data during the pandemic, without distinction between quarantined and non-quarantined periods.

The training frequency among the athletes surveyed showed a statistically significant drop in the pre-pandemic period and during the pandemic. This situation presents similar behavior in the literature, and the average results of studies analyzed in a meta-analysis shows a similar frequency behavior in both periods (20). The study sample showed significant training type changes for strength and cardio training, with both seeing fewer days. Sadly, this data point cannot be compared, since no published evidence was found under these parameters; however, it aligns with the weekly frequency.

Training times showed a coincidence between the behavior of the local sample and athletes in other countries, with a decrease in training sessions' duration during PWQ to half as much as PP sessions. In line with these results, Pillay et al. reported sessions lasting under an hour (21) which is unusually short for elite athletes; the same behavior arose with athletes in other disciplines (18). It would be interesting in the future to associate these data with the presence of distractors in the athletes' homes, or with the difficulty in distributing the work-sport-homework times in the daily routine during the confinement since these factors could have been determinants in the decrease in the frequency of training.

Training intensity fell during PWQ compared with the PP period in our study sample, which aligns with other reports (18, 21) (even when athletes surveyed by these authors were from elite and semi-elite groups). It´s possible to theorize that this phenomenon was due to the fact that many athletes, at least at the beginning of the confinement, did not have the necessary equipment and space in their homes to maintain the level of training they had before the pandemic, with a consequent and involuntary reduction in workload.

Before the pandemic, urban outdoor locations were the main training space, similar to PWOQ. As noted previously, the present study considered three timeframes, making it reasonable to expect that during PWQ the majority (70.06%) declared that their homes were their main training location. A Norwegian study declared that using public open spaces as an area for practicing open-air physical activities rose by 291% compared with PP (22).

However, in the present study, it was observed that outdoor training remained similar between the PP and PWOQ periods, both overall and in the individual disciplines.

Various studies confirm that during lockdowns, incorporating technology became a fundamental tool for evaluating training via different monitoring devices, such as watches or applications, specific platforms with virtual trainers (either synchronous or asynchronous), and others (15, 23, 24). Virtual environments also provide great opportunities to compete and socialize amongst other athletes during training (13, 15).

Athletes' sociodemographic characteristics placed them mainly within higher educational levels, which probably helped them seek out and use the technological tools to continue their disciplines even under adverse conditions.

To the authors' opinion It would be important to investigate more about the type of virtual monitoring related with cardiovascular risk and relative injury risk when training while using devices, in order to guarantee safe training from a health perspective.

Both the monthly training budget and the amount invested in equipment in the PP, PWQ and PWOQ periods did not show significant differences among athletes in general, nor by discipline.

The main limitation of this study is the survey methodology. The majority of available studies about changes faced by athletes during the COVID-19 pandemic used self-administered surveys via online platforms like Google or social media applications, as with the present study (4, 14, 18, 21, 25). While this methodology facilitated access to the sample during quarantine or lockdown periods, the instruments' self-administered character could present a participation bias, since every respondent can include or exclude themselves from participating in the study, or else give partial responses to each survey, limiting the validity of the information gathered. However, the methodology of evaluation through self-report questionnaires for physical activity has been on the rise since the pandemic (26). Another limitation to consider is that sampling introduces volunteer bias. So, the results should be interpreted with caution, as the sample does not necessarily represent the universe of athletes in Chile.

Even considering these limitations, the authors consider that this study is a contribution to knowledge in the area, and its methodology could be useful for use in health or other crisis.

## Conclusions

All variables related with training habits fell when comparing the PP and PWQ periods, making it logical to indicate that there was more training taking place before the pandemic. All the variables rose between PWQ and PWOQ. However, comparing the PP and PWOQ periods, there are very slight differences, and not always in favor of PWOQ, which reflects how the athletes have still not been able to return to their training rhythm.

Among the disciplines analyzed, runners saw the most alterations in their training routines, directly affecting frequency, type, and time. The variables show a decrease when comparing PP and PWQ, and the values in PWOQ approach those declared in PP.

Triathletes generally reported no significant differences between periods. However, they showed a notorious trend towards greater frequency and time when comparing PP and PWOQ. Cyclists' overall behaviors align with the general results.

Finally, we can also conclude that the use of technology increased for training and monitoring during and after lockdowns. As the use of technology has become more widespread, independent of the health crisis that prompted this study, future research should investigate the type of virtual monitoring related to cardiovascular risk and relative injury risk when training while using devices, to guarantee safe training from a health perspective.

## Data Availability

The raw data supporting the conclusions of this article will be made available by the authors, without undue reservation.

## Appendix 1

### SURVEY: Impact of the COVID-19 pandemic on training habits and technology use among Chilean amateur athletes

Identification: email

1. Dimension: Sociodemographic data

**Table:**

Variable	Question	Response
Age	What is your birth date?	DD/MM/YYYY
Sex	What is your biological sex?	Female Male Other
Education Level (EDL)	What is your highest completed education level?	No formal schooling Primary, incomplete Primary school graduate Secondary technical or scientific/humanist, incomplete Humanities/arts, incomplete Secondary technical or scientific/humanist graduate Humanities/arts graduate Technical institute (CFT) or professional institute, incomplete (1–3 year programs) Technical institute (CFT) or professional institute graduate (1–3 year programs) / sub-officer in Armed Forces/Police Undergraduate, incomplete (programs of 4 years or more) University graduate (programs of 4 years or more) / officer in Armed Forces /Police Postgraduate (master’s, doctor)
Socioeconomic level	What is your total monthly household income? Consider income from the work of all people in your household (pensions, dividends, rents, family contributions, and any other income)	AB (avg.: CLP7,177,530) C1a (avg.: 3,010,391) C1b (avg.: 2,072,853) C2 (avg.: 1,500,774) C3 (avg.: 1,003,426) D (avg.: 640,667) E (avg.: 361,583)
National region	What region did you live in during the pandemic?	I to XV
Municipality	What town did you live during the pandemic?	Complete
Origin	Your home during the pandemic is located in a ___ zone:	Urban Rural
Occupation	In your main occupation, you work as a:	Employer or owner Self-employed worker Public-sector employee or worker (includes municipalities) Public enterprise employee or worker Private-sector employee or worker Domestic service Unpaid family work Armed forces and police Housewife Unemployed
Sport Type	What sport do you take part in the most often?	Running Cycling Swimming Triathlon (if you are a triathlete, only mark this option, and not all the others separately)
Time spent on sports as a regular routine	How long have you been doing your training routine in a systematic way?	Complete (years + months)

2. Dimension: Training habits

Answer the following questions about your training habits, considering how they were before the start of the COVID-19 pandemic in Chile (March 2020); during quarantine periods in your city (total movement restrictions) ; and during non-quarantine periods in your city (partial movement restriction and participation according to permitted occupancy).

**Table:**

What is your…?	Pre-Pandemic	Periods WITH Quarantine (Phase 1)	Periods WITHOUT Quarantine (Phase 2, 3, 4)
Average training frequency (times/week)	___times/week	___ times/week	___ times/week
Average weekly training duration (minutes/week)	___minutes/week	___ minutes/week	___ minutes/week
Number of days per week with HIGH intensity training (80%–90% MHR or >85% 1MR)	____ days/week	____ days/week	____ days/week
Number of days per week with MODERATE intensity training (70%–80% MHR or 60%–85% 1MR)	____ days/week	____ days/week	____ days/week
Number of days per week with LOW intensity training (60%–70% MHR or 30%–60% 1MR)	____ days/week	____ days/week	____ days/week
Main training site: (you can mark more than one option) - Home (1) - Gym (2) - Urban outdoors (3) - Nature (4)			
Number of days per week with CARDIO training	____ days/week	____ days/week	____ days/week
Number of days per week with STRENGTH training	____ days/week	____ days/week	____ days/week

3. Dimension: Training Budget

How much do you spend for training, on average?

Amount in pesos ($CLP) spent on MONTHLY training considering:

**Table:**

Budget portion for:	Pre-Pandemic	Periods WITH Quarantine (Phase 1)	Periods WITHOUT Quarantine (Phase 2, 3, 4)
Gym	Fill in	Fill in	Fill in
Trainer	Fill in	Fill in	Fill in
Health professionals	Fill in	Fill in	Fill in

Amount in pesos ($CLP) spent on training equipment during 2019–2021, including:

**Table:**

Budget portion for:	Pre-Pandemic	Periods WITH Quarantine (Phase 1)	Periods WITHOUT Quarantine (Phase 2, 3, 4)
Monitoring Devices	Fill in	Fill in	Fill in
Machinery-equipment	Fill in	Fill in	Fill in
Training software	Fill in	Fill in	Fill in
Sportswear	Fill in	Fill in	Fill in
Nutritional supplements	Fill in	Fill in	Fill in

4. Dimension: Technology use

Answer the following questions related with implementing, planning, and monitoring your training sessions considering how they were before the start of the COVID-19 pandemic (March 2020); during quarantine periods in your city (total movement restriction); and during periods without quarantine in your city (partial movement restriction and participation according to permitted occupancy).

**Table:**

During your training sessions, did you:	Pre-Pandemic	Periods WITH Quarantine (Phase 1)	Periods WITHOUT Quarantine (Phase 2, 3, 4)
Use devices to measure your training parameters	___No ___ Yes _______ What	___No ___ Yes _______ What	___No ___ Yes _______ What
Use equipment to perform training	___No ___ Yes _______ What	___No ___ Yes _______ What	___No ___ Yes _______ What
Use specialized software to perform training	___No ___ Yes _______ What	___No ___ Yes _______ What	___No ___ Yes _______ What

## Appendix 2. Invitation to expert judges for Survey Validation process

You are cordially invited to participate in the validation process of a survey as a data-gathering instrument in the framework of the research project **“Impact of the COVID-19 pandemic on training and technology use among Chilean amateur athletes”**, which is being performed by a team in the Physical Therapy School at Universidad Mayor. This manuscript is also part of the thesis work within the context of the Doctoral Program in Biomedical Research Methodology and Public Health.

The objective of the study is to evaluate the impact of the COVID-19 pandemic on the training habits of Chilean amateur runners, cyclists, swimmers, and triathletes. To this end, training habits will be considered from 3 timeframes: prior to the COVID-19 pandemic, during the pandemic with quarantine, and during the pandemic but without quarantine. The proposal intends to characterize the sample of athletes from a sociodemographic perspective, comparing training level by sport type, quantify training modifications, identify different practice modes, technological tool use, budget variations, and the influence of movement restrictions due to the pandemic upon training motivation. These variables will be analyzed across the three aforementioned timeframes.

To fulfill the presented objectives, a 44-question brief-response survey was drafted with quantitative and qualitative indicators, which in turn aid with comprehending 5 dimensions:

(1) Sociodemographic data.
(2) Training habits
(3) Training budget
(4) Technology use
(5) Motivation.

We request an evaluation only for the dimensions regarding **training habits, training budget, and technology use**, since the sociodemographic data will only have a descriptive analysis, and to evaluate the motivational dimension we will use the Sport Motivation Scale-2 (SMS-2), validated for the Chilean population in 2013.

In order to guarantee that the instrument is suitable and valid in its content, we request your participation in the content evaluation process via the “consult an external expert judge panel” modality. In this context, we would like to invite you to join this panel, since your knowledge, your work area, and your expertise allow you to offer highly valuable judgment.

**Instructions:**

1. We request that you review the questions and possible response options, and make comments to improve comprehension of the instrument, if you deem it pertinent in your expert opinion.
2. For comprehensive effects, a **dimension or domain** will be understood as a global area or unit to explore via the data gathering instrument (in this case, there are three: training habits, training budget, and technology use).
3. The validity of each item or question will be evaluated via 3 categories: Essential, useful but expendable, unnecessary.
4. **Concerning sufficiency**, unlike the previous, evaluation cannot be by item, but rather by groups of items comprising the dimension to be evaluated, given that what is under consideration is whether they are sufficient for the purpose or not. Each category must be quantified with an indicator, with values from 1 to 4, as the next table shows:

Content validity evaluation criteria for external expert judges^6^.

**Table:**

Categories	Indicators
Sufficiency(*) Items belonging to the same dimension are sufficient to obtain measurement of this one	1. Items are insufficient to measure the dimension 2. Items measure some aspect of the dimension, but do not correspond to the entire dimension 3. Some items must be increased to be able to fully evaluate the dimension 4. The items are sufficient.

## References

[1] Guo YR Cao QD Hong ZS Tan YY Chen SD Jin HJ The origin, transmission and clinical therapies on coronavirus disease 2019 (COVID-19) outbreak—an update on the status. Mil Med Res. (2020) 7(1):11. 10.1186/s40779-020-00240-0 PMC7068984 32169119

[2] Bok D Chamari K Foster C. The pitch invader-COVID-19 canceled the game: what can science do for US, and what can the pandemic do for science? Int J Sports Physiol Perform. (2020) 15:917–9. 10.1123/ijspp.2020-0467 32570215

[3] Jagim AR Luedke J Fitzpatrick A Winkelman G Erickson JL Askow AT The impact of COVID-19-related shutdown measures on the training habits and perceptions of athletes in the United States: a brief research report. Front Sports Act Living. (2020) 2:1–6. 10.3389/fspor.2020.623068 PMC7785865 33426521

[4] Guilherme FR Do Nascimento MA Fiorillo RG da Silva MC Amadeu GDS Graça Á Perceptive changes in endurance athletes during social isolation due to COVID-19. Rev Bras Med Esporte. (2020) 26(6):473–7. 10.1590/1517-8692202026062020_0072

[5] Amateur vs. professional athletes. USLegal. United States.

[6] AlSamhori JF Alshrouf MA AlSamhori ARF Alshadeedi FM Madi AS Alzoubi O. Implications of the COVID-19 pandemic on athletes, sports events, and mass gathering events: review and recommendations. Sports Med Health Sci. (2023) 5:165–73. 10.1016/j.smhs.2023.07.006 37753427 PMC10518794

[7] Holmes HH Monaghan PG Strunk KK Paquette MR Roper JA. Changes in training, lifestyle, psychological and demographic factors, and associations with running-related injuries during COVID-19. Front Sports Act Living. (2021) 3:1–14. 10.3389/fspor.2021.637516 PMC8215167 34164619

[8] Segui-Urbaneja J Julião RP Manuel R Mendes N Dorado V Farías-Torbidoni EI. The impact of COVID-19 on physical activity on people who participate on running and cycling sporting events people in Spain and Portugal. Available online at: www.retos.org

[9] Chan ZYS Peeters R Cheing G Ferber R Cheung RTH. Evaluation of COVID-19 restrictions on distance runners’ training habits using wearable trackers. Front Sports Act Living. (2022) 3:812214. 10.3389/fspor.2021.812214 35098124 PMC8790471

[10] Fiorilli G Grazioli E Buonsenso A Di Martino G Despina T Calcagno G A national COVID-19 quarantine survey and its impact on the Italian sports community: implications and recommendations. PLoS One. (2021) 16(3):e0248345. 10.1371/journal.pone.0248345 33720968 PMC7959356

[11] Giulianotti R Chtourou H Driss T Serrano-Rosa MA Moreno-Tenas A León-Zarceño E. The Use of Online Training Tools in Competition Cyclists During COVID-19 Confinement in Spain (2021). Available online at: www.frontiersin.org

[12] Tjønndal A. #Quarantineworkout: the use of digital tools and online training among boxers and boxing coaches during the COVID-19 pandemic. Front Sports Act Living. (2020) 2:589483. 10.3389/fspor.2020.589483 PMC7739676 33345163

[13] DeJong AF Fish PN Hertel J. Running behaviors, motivations, and injury risk during the COVID-19 pandemic: a survey of 1147 runners. PLoS One. (2021) 16(2):e0246300. 10.1371/journal.pone.0246300 PMC7880469 33577584

[14] Parm Ü Aluoja A Tomingas T Tamm AL. Impact of the COVID-19 pandemic on estonian elite athletes: survey on mental health characteristics, training conditions, competition possibilities, and perception of supportiveness. Int J Environ Res Public Health. (2021) 18(8):4317. 10.3390/ijerph18084317 33921723 PMC8074017

[15] Parra-Camacho D Alonso Dos Santos M González-Serrano MH. Amateur runners’ commitment: an analysis of sociodemographic and sports habit profiles. Int J Environ Res Public Health. (2020). 17(3):925. 10.3390/ijerph17030925 32024280 PMC7037282

[16] Transición Cuarentena P. Apertura Avanzada Apertura inicial Estrategia Gradual 2020. Available online at: www.gob.cl/coronavirus/pasoapaso/

[17] Galicia Alarcón L Balderrama Trapaga J Edel Navarro R. Validez de contenido por juicio de expertos: propuesta de una herramienta virtual. Apertura: Revista de Innovación Educativa. (2017) 9(2):42–53. 10.32870/ap.v9n2.993

[18] Esht V. Impact of the COVID-19 pandemic on physical. Psychological and nutritional characteristics of elite athletes: a cross-sectional web survey. Biosci Biotechnol Res Commun. (2021) 14(1):203–8. 10.21786/bbrc/14.1/29

[19] Cohen J. Statistical Power Analysis for the Behavioral Sciences. 2nd edn. New York: Lawrence Erlbaum Associates, Publishers (1988).

[20] Brand R Nosrat S Späth C Timme S. Using COVID-19 pandemic as a prism: a systematic review of methodological approaches and the quality of empirical studies on physical activity behavior change. Front Sports Act Living. (2022) 4:1–17. 10.3389/fspor.2022.864468/full PMC9069113 35529420

[21] Pillay L Janse van Rensburg DCC Jansen van Rensburg A Ramagole DA Holtzhausen L Dijkstra HP Nowhere to hide: the significant impact of coronavirus disease 2019 (COVID-19) measures on elite and semi-elite South African athletes. J Sci Med Sport. (2020) 23(7):670–9. 10.1016/j.jsams.2020.05.016 32448749 PMC7235602

[22] Venter ZS Barton DN Gundersen V Figari H Nowell M. Urban nature in a time of crisis: recreational use of green space increases during the COVID-19 outbreak in Oslo, Norway. Environ Res Lett. (2020) 15(10):104075. 10.1088/1748-9326/abb396

[23] Adrian J Abdulaziz W Isabel F David K Evert BP. Training during the COVID—19 lockdown : knowledge, beliefs, and practices of 12, 526 athletes from 142 countries and six continents. Sports Medicine. (2022) 52(4):933–48. 10.1007/s40279-021-01573-z 34687439 PMC8536915

[24] Janssen M Walravens R Thibaut E Scheerder J Brombacher A. Understanding different types of recreational runners and how they use running-related technology. Int J Environ Res Public Health. (2020) 17(7):2276. 10.3390/ijerph17072276 32230999 PMC7177805

[25] Tingaz EO. The psychological impact of COVID-19 pandemic on elite athletes, management strategies and post-pandemic performance expectations: a semi structured interview study. Int J Educ Res Innov. (2020) 15(15):73–81. 10.46661/ijeri.4863

[26] Nigg CR Fuchs R Gerber M Jekauc D Koch T Krell-Roesch J Assessing physical activity through questionnaires—a consensus of best practices and future directions. Psychol Sport Exerc. (2020) 50(October 2019):101715. 10.1016/j.psychsport.2020.101715

